# Low- Versus High-Concentration Iodine Contrast for Hepatic Multiphase CT in Chronic Liver Disease: Image Quality, Lesion Detectability, and Iodine Load Reduction with Modern MDCT—A Retrospective Non-Inferiority Study

**DOI:** 10.3390/diagnostics15233026

**Published:** 2025-11-27

**Authors:** Bo Kyung Kim, Jin Sil Kim, Hyo Jeong Lee, Jeong Kyong Lee, Hye Ah Lee, Seongyong Pak

**Affiliations:** 1 Department of Radiology, College of Medicine, Ewha Womans University, Seoul 07985, Republic of Korea; 251meg01@ewha.ac.kr (B.K.K.); hjleerad@ewha.ac.kr (H.J.L.); kyongmd@ewha.ac.kr (J.K.L.); 2 Clinical Trial Center, Mokdong Hospital, Ewha Womans University, Seoul 07985, Republic of Korea; khyeah@ewha.ac.kr; 3 Department of Biomedical Engineering, Asan Medical Institute of Convergence Science and Technology, Asan Medical Center, University of Ulsan College of Medicine, Seoul 05505, Republic of Korea; seongyong.pak@siemens-healthineers.com

**Keywords:** hepatic multiphase CT, low-concentration iodine contrast, image quality, lesion detectability, tube voltage

## Abstract

**Background/Objectives**: Lower iodine concentration may mitigate nephrotoxicity by decreasing osmolality and viscosity. With modern multidetector CT (MDCT) and hybrid iterative reconstruction, reducing total iodine load without compromising image quality is feasible. We evaluated whether hepatic multiphase CT using low-concentration iodine contrast (LCIC, 270 mg I/mL) provides non-inferior image quality and lesion detectability compared with high-concentration iodine contrast (HCIC, 350 mg I/mL), and we identified iodine dose thresholds for acceptable image quality. **Methods**: We retrospectively analyzed 179 HCIC and 190 LCIC multiphase CT examinations. Arterial-phase imaging used 100 or 120 kVp; the portal-venous phase used automated tube-voltage modulation. We prespecified a non-inferiority margin of −0.5 for the mean image-quality score. Image quality and diagnostic performance were compared, and iodine dose thresholds for acceptable quality were determined using AUC analysis. **Results**: Arterial-phase image-quality scores were 4.450 ± 0.462 (HCIC) versus 4.439 ± 0.477 (LCIC) (difference, −0.010; 95% CI, −0.107 to 0.086). Portal-venous scores were 4.430 ± 0.443 and 4.337 ± 0.371, respectively (difference, −0.093; 95% CI, −0.177 to −0.010). Both met non-inferiority. Per-patient diagnostic performance was comparable (0.931 versus 0.947; *p* = 0.38). Per-lesion detectability was also similar (0.862 versus 0.909; *p* = 0.18), whereas per-lesion diagnostic performance differed (0.669 versus 0.781; *p* = 0.02). Optimal iodine dose thresholds were 501.691 mg I/kg at 100 kVp and 599.145 mg I/kg at 120 kVp for the arterial phase, and 517.650 mg I/kg for the portal-venous phase. **Conclusions**: LCIC hepatic multiphase CT provided non-inferior image quality and diagnostic performance compared with HCIC on contemporary MDCT with hybrid iterative reconstruction. The iodine dose required to preserve image quality varied by tube voltage, supporting tailored protocols.

## 1. Introduction

Multiphase liver CT is central to detecting and staging liver disease—particularly hepatocellular carcinoma (HCC)—in at-risk patients who require long-term imaging surveillance. Historically, high-concentration iodine contrast media (more than 350 mg I/mL) have been used to ensure adequate image quality and enhancement for lesion detection [1]. Liver CT enhancement depends on iodine dose and on the interplay of patient factors (e.g., body weight, cardiac output), contrast parameters (e.g., iodine concentration, volume, injection rate), and acquisition settings (e.g., tube voltage, reconstruction technique) [2,3,4,5]. With the adoption of advanced MDCT systems and iterative reconstruction, it has become feasible to reduce iodine load without degrading image quality.

Earlier studies using 64-channel MDCT primarily sought the optimal iodine dose for HCC detection [1,6]. Most dose-reduction strategies decreased injection volume, whereas few explored the clinical feasibility of lowering iodine concentration itself [1,3,5,6,7]. Lowering contrast concentration reduces osmolality and viscosity, which can limit intratubular pressure and preserve renal perfusion, thereby lowering the likelihood of kidney injury [8,9,10]. Additionally, low-concentration iodine contrast (LCIC) requires lower injection pressures, reducing the risk of extravasation and improving hemodynamic tolerance, particularly in patients with cardiovascular comorbidities [2,11]. Moreover, recent developments such as deep learning-based reconstruction and dual-energy CT have shown promise in preserving diagnostic performance at low dose [12,13,14]. These advances support re-evaluation of conventional contrast protocols. However, in clinical settings without such advanced technologies, it remains important to determine whether LCIC can maintain diagnostic image quality in patients with chronic liver disease when using modern (≥64-channel) MDCT with iterative reconstruction.

Therefore, we investigated whether hepatic multiphase CT using LCIC (270 mg I/mL) provides non-inferior image quality and diagnostic performance compared with HCIC (350 mg I/mL) in patients with chronic liver disease. We also emphasized arterial-phase imaging at different tube voltages (100 versus 120 kVp) to determine optimal iodine dose thresholds and to identify opportunities for protocol optimization.

## 2. Materials and Methods

### 2.1. Patient Population

This retrospective study was approved by our institutional review board; Ewha Womans University Mokdong Hospital (approval code 2024-09-012). The low-concentration iodine contrast agent used in this study is FDA-approved. Prior to protocol transition, internal validation confirmed that image quality and diagnostic confidence were not affected. Two abdominal radiologists reviewed the results using deep learning-based reconstruction and monoenergetic (mono-keV) imaging. Based on these validations, the institution formally adopted the low-concentration protocol. The IRB approved the retrospective study but not the clinical protocol change, which was considered a standard optimization. This study was conducted independently, without any control or influence from the company over its design, conduct, data analysis, or reporting.

**Figure 1:**
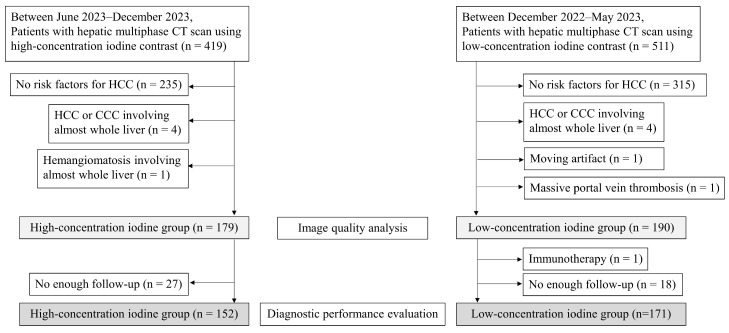
Patient enrollment flowchart.

Between December 2022 and May 2023, 419 patients underwent hepatic multiphase CT using HCIC (350 mg I/mL; Iobitridol [Xenetix 350, Guerbet, Sulzbach, Germany], Iohexol [Bonorex 350, Central Medical Service, Seoul, Republic of Korea]). After excluding patients without HCC risk factors according to LI-RADS 2018 [15] (*n* = 235), those with HCC or cholangiocarcinoma (CCC) involving almost the entire liver (*n* = 4), and those with hemangiomatosis involving almost the entire liver (*n* = 1), 179 patients who underwent HCIC CT were included for image-quality analysis. Between June 2023 and December 2023, 511 patients underwent hepatic multiphase CT using LCIC (270 mg I/mL; Iohexol [Iobrix inj. 270; Taejoon Pharm Co., Seoul, Republic of Korea]). After excluding patients without HCC risk factors according to LI-RADS 2018 [15] (*n* = 315), those with HCC or CCC involving almost the entire liver (*n* = 4), those with severe motion artifact (*n* = 1), and those with massive portal vein thrombosis (*n* = 1), 190 patients who underwent LCIC CT were included for image-quality analysis (Figure 1).

This study was designed as an extension of our previous work [14], which evaluated the performance of a low-concentration contrast agent in monoenergetic (mono-keV) im-aging. Overall, 39 patients overlapped in both studies. However, the previous study analyzed monoenergetic images, whereas the current study analyzed mixed images reconstructed to simulate single-energy acquisition. The current analysis focused on diagnostic performance under single-energy (single-kVp) conditions using different contrast concentrations across a larger cohort. To minimize temporal bias, all scans were obtained within the shortest possible interval and performed on the same CT system. The total iodine dose at each tube voltage (kVp) was also compared to provide practical guidance for protocol optimization.

### 2.2. Image Acquisition and Reconstruction

Imaging protocols were identical between groups, differing only in contrast agent. All scans used either a CT scanner (SOMATOM Definition Flash; Siemens Healthcare, Forchheim, Germany; *n* = 172; HCIC CT, *n* = 138; LCIC CT, *n* = 34) or a 192-detector-row CT scanner (SOMATOM Force; Siemens Healthcare, Forchheim, Germany; *n* = 197; HCIC CT, *n* = 41; LCIC CT, *n* = 156) with automated tube current modulation (CareDose4D, Siemens Healthcare). Arterial-phase images were obtained in dual-energy (DE) mode. For the SOMATOM Definition Flash, parameters were 32 × 0.6 mm collimation, pitch 0.6, 3 mm reconstruction interval, and two tube voltages (100 kV and tin-filtered 140 kV [Sn140 kV]) with reference tube currents of 219/170 mAs. For scans on the SOMATOM Force, acquisition parameters were 128 × 0.6 mm collimation, pitch 0.6, 3 mm reconstruction interval, and two tube voltages (80 kV and tin-filtered 150 kV [Sn150 kV]) with reference tube currents of 325/163 mAs. To enable comparison with single-energy arterial-phase images, we generated blended images from dual-energy acquisitions. For the SOMATOM Definition Flash, DE scans were reconstructed into blended images with a 0.5 mixing ratio (50% 100 kV, 50% Sn140 kV), approximating a 120 kVp image (Supplementary Figure S1). For the SOMATOM Force, mixed images used a 0.6 ratio (60% 80 kV, 40% Sn150 kV), approximating a 100 kVp image (Supplementary Figure S2). These blended dual-energy images closely resembled single-energy acquisitions at the corresponding tube potentials, as illustrated in Supplementary Figures S1 and S2, which visually demonstrate the equivalence of attenuation and image appearance between the dual- and single-energy modes. All mixed images were reconstructed with the ADMIRE 3 model-based iterative reconstruction algorithm, a partial model-based technique reported to yield high-quality abdominal CT images [16].

Unenhanced scans were acquired first. Contrast agent (HCIC or LCIC) was dosed by body weight: 110 mL for patients ≤ 60 kg and 120 mL for those >60 kg (approximately 600 mg I/kg for a 70 kg individual for HCIC CT and 463 mg I/kg for a 70 kg individual for LCIC CT, respectively). Injection was performed at 3 mL/s using an automatic power injector. Arterial-phase acquisition was triggered 12 s after a 100-HU threshold was reached in the abdominal aorta using bolus tracking. Portal venous and delayed-phase images were obtained with single-energy (SE) scans and automated tube-voltage modulation (CARE kV). For the SOMATOM Definition Flash, parameters were 128 × 0.6 mm collimation, pitch 0.8, 3 mm reconstruction interval, reference tube voltage 120 kV, and reference current 150–180 mAs. For the SOMATOM Force, acquisition parameters were 192 × 0.6 mm collimation, pitch 0.6, 3 mm reconstruction interval, reference tube voltage 120 kV, and reference tube current 150 mAs. All SE images were reconstructed using the ADMIRE 3 model-based iterative reconstruction algorithm. Portal venous-phase images were acquired 80–90 s after contrast injection, and delayed-phase images at 180 s. The portal venous phase covered the lower chest through the pelvic cavity, whereas the other phases extended from the lower chest to the inferior liver margin.

### 2.3. Qualitative Image Analysis

Qualitative image quality in the arterial and portal venous phases was assessed independently by two radiologists (J.S.K. and J.K.L.), both blinded and both formally trained in LI-RADS interpretation, with 13 years (J.S.K.) and more than 20 years (J.K.L.) of experience in abdominal CT. Both reviewers were blinded to the contrast agent used for each scan. All assessments were performed using the institutional picture archiving and communication system (PACS; INFINITT Healthcare, Seoul, Republic of Korea). Radiologists could freely adjust window width and level to optimize visualization. Overall image quality in the arterial and portal venous phases was graded on a 5-point Likert scale as follows: 1, very poor (nondiagnostic; re-examination required); 2, suboptimal (insufficient but interpretable); 3, moderate (acceptable with noticeable limitations); 4, good (adequate for confident clinical interpretation); and 5, excellent (optimal image quality for diagnosis) [17]. Hepatic-artery clarity on arterial-phase images was also assessed on a dedicated 5-point scale: 1, very poor (not delineated); 2, suboptimal (barely distinct); 3, moderate (common and proper hepatic arteries visible; segmental arteries blurred); 4, above average (bilateral hepatic arteries clearly visible; segmental branches blurred); and 5, excellent (clear visualization of second-order hepatic-artery branches). Liver parenchymal contrast in the portal venous phase was also evaluated on a 5-point scale: 1, very poor (minimal contrast enhancement, resembling unenhanced or nephrogenic-phase images); 2, suboptimal (poor parenchymal enhancement); 3, moderate (adequate but not strong contrast); 4, good parenchymal enhancement; and 5, excellent liver enhancement with clearly defined parenchymal detail.

### 2.4. Quantitative Image Analysis

Quantitative analyses, including signal-to-noise ratio (SNR) and contrast-to-noise ratio (CNR), were conducted by a single radiologist (B.K.K., with four years of experience in abdominal imaging). For liver SNR, circular regions of interest (ROIs; approximately 1–3 cm^2^) were placed in homogeneous parenchyma in four segments (right anterior, right posterior, left medial, left lateral) at the portal vein level on arterial- and portal-phase images. Liver parenchymal noise (noise_liver_) was defined as the mean standard deviation (SD) of Hounsfield units across the four ROIs. Liver SNR was calculated as mean liver attenuation divided by the corresponding noise: SNR_liver_ = mean HU_liver_/noise_liver_ [18].

For the CNR evaluation of HCC, lesions were included if at least one reviewer visually detected them; CNR was calculated separately for the arterial and portal venous phases. Lesions not detectable in a given phase were excluded from the CNR calculation for that phase. A freehand ROI was drawn to encompass as much of the lesion as possible. Each lesion was measured three times, and the mean was used. CNR for each lesion was calculated as: CNR_lesion_ = (mean HU_lesion_ − mean HU_liver_)/image noise. CNRs for the aorta (arterial phase) and portal vein (portal phase) were also evaluated using: CNR of the aorta = (mean HU of the aorta − mean HU of the subcutaneous fat)/image noise; CNR of the portal vein = (mean HU of the portal vein − mean HU of the subcutaneous fat)/image noise [19]. Image noise was defined as the standard deviation of HU within an ROI in subcutaneous fat and served as the denominator for vascular and parenchymal CNRs.

### 2.5. Diagnostic Performance and Focal Liver Lesion Evaluation

Diagnostic performance and focal lesion analyses were independently conducted by two board-certified radiologists (J.S.K., with 13 years of experience; and J.K.L., with more than 20 years of experience in abdominal CT interpretation), both of whom also participated in the qualitative assessment. For diagnostic performance, patients were eligible if they met one of the following: (1) focal liver lesions confirmed malignant by additional diagnostics or imaging, or (2) benign lesions or no lesions with ≥12 months of adequate imaging follow-up. Patients lacking pathologic confirmation or sufficient imaging follow-up were excluded for lack of a reference standard. Reference standards were established by comprehensive review of clinical records, including electronic medical records, surgical or biopsy pathology results, and imaging (CT, MRI, or PET/CT). Based on these criteria, a total of 323 patients were finally included in the diagnostic performance analysis (HCIC, *n* = 152; LCIC, *n* = 171) (Figure 1). Per-patient diagnostic accuracy was assessed; among these patients, those with focal liver lesions underwent per-lesion analyses of diagnostic accuracy and detectability. Typical wedge-shaped arterioportal shunts and simple cysts were excluded. The two reviewers independently rated lesion conspicuity on a 5-point scale (1, not distinct; 2, barely distinct; 3, moderately distinct; 4, fairly distinct; 5, definitely distinct). Scores of 3–5 were considered detected, and scores of 1–2 were considered non-detected. Conspicuity was defined as the higher score across the arterial and portal venous phases. Each lesion was assigned a Liver Imaging Reporting and Data System (LI-RADS) category [15]. LR-4 or LR-5 were interpreted as indicative of hepatocellular carcinoma (HCC), and LR-M as another hepatic malignancy, such as adenocarcinoma.

### 2.6. Statistical Analysis

Statistical significance was set at two-tailed *p* < 0.05. Analyses were performed using IBM SPSS Statistics (v29.0 [IBM, Armonk, NY, USA]), MedCalc (v19.2.1 [MedCalc, Marikerke, Belgium]), and R software (v4.3.3 [R Foundation for Statistical Computing, Vienna, Austria]). Categorical variables were compared using the chi-square (χ^2^) test. Continuous variables were analyzed using Student’s *t*-test or Mann–Whitney U test, as appropriate. Interobserver agreement for qualitative assessments was evaluated using Gwet’s agreement coefficient (AC2) calculated with the irrCAC package in R [20]. AC2 was interpreted using standard thresholds [21]: ≤0.20, poor; 0.21–0.40, fair; 0.41–0.60, moderate; 0.61–0.80, good; 0.81–1.00, very good.

The primary endpoint was to determine whether LCIC CT was non-inferior to HCIC CT for overall image quality in arterial and portal phase images. A non-inferiority margin of −0.5, derived from previously published studies [12,14,22,23], was predefined. Both arterial and portal-venous phases were pre-specified as co-primary endpoints; therefore, no additional multiplicity adjustment was applied. Non-inferiority was concluded when the lower bound of the 95% confidence interval (equivalent to the one-sided 97.5% interval) for the mean difference (LCIC − HCIC) exceeded the predefined margin (−0.5). Receiver operating characteristic (ROC) analysis identified iodine dose cutoffs associated with a mean image-quality score more than 4 in both phases. The optimal weight-normalized iodine dose was determined by maximizing the Youden index (sensitivity + specificity − 1), which identifies the ROC point that best separates diagnostically acceptable from suboptimal image quality. Per-patient diagnostic accuracy was evaluated using generalized estimating equations (GEE) with a binomial distribution and a logit link [19]. A multivariable GEE model assessed the association between contrast group and diagnostic accuracy, adjusting for voltage, presence of ascites, portal hypertension, and body mass index. Per-lesion diagnostic performance and detectability were also evaluated using GEE. Subgroup analyses were performed by body mass index (BMI < 25 versus ≥25 kg/m^2^) and lesion size (<20 mm versus ≥20 mm). Reader-averaged figures of merit (FOMs) were calculated using weighted jackknife alternative free-response receiver operating characteristic (JAFROC) analysis with fixed-reader, random-lesion modeling in JAFROC (v4.1).

## 3. Results

### 3.1. Patient Population

In total, 369 patients were analyzed: 179 in the HCIC group and 190 in the LCIC group. No statistically significant differences were observed between groups in age, sex, body weight, body mass index, chronic liver disease etiology, or baseline liver and renal function (Table 1). The mean contrast amount differed significantly between groups (626.189 ± 124.592 versus 468.697 ± 74.053; *p* < 0.001).

### 3.2. Qualitative Analysis

**Figure 2:**
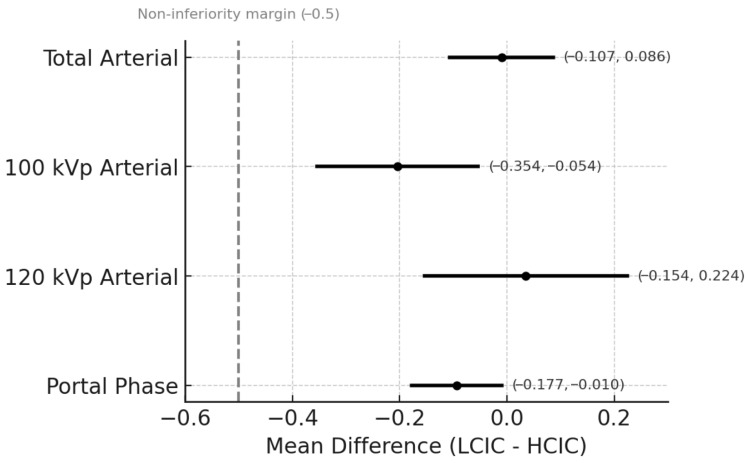
Non-inferiority analysis of overall image quality comparing low-concentration iodine contrast CT (LCIC CT) with high-concentration iodine contrast CT (HCIC CT). Mean differences in overall image quality with 95% confidence intervals (CIs) are shown for the total arterial phase, the arterial phase at 100 kVp and 120 kVp, and the portal venous phase. The predefined non-inferiority margin of −0.5 is denoted by the dotted vertical line. Across all settings, the lower bound of the 95% CI exceeds this margin, supporting non-inferiority of LCIC CT compared with HCIC CT.

Subjective arterial-phase overall image quality was high for both groups (HCIC, 4.450 ± 0.462; LCIC, 4.439 ± 0.477), demonstrating that LCIC was non-inferior (mean difference, −0.010; 95% CI, −0.107 to 0.086). Hepatic artery clarity in the arterial phase was similar between groups (HCIC, 4.802 ± 0.380; LCIC, 4.834 ± 0.357), supporting LCIC CT non-inferiority (mean difference, 0.033; 95% CI, −0.043 to 0.108). In the portal phase, overall image quality (4.430 ± 0.443 versus 4.337 ± 0.371) and liver contrast score (4.536 ± 0.407 versus 4.384 ± 0.391) were significantly lower in the LCIC group than in the HCIC group; however, both measures met the predefined non-inferiority margin of −0.5 (mean difference, −0.093; 95% CI, −0.177 to −0.010; and −0.152; 95% CI, −0.234 to −0.070, respectively) (Table 2 and Figure 2). Interobserver agreement, assessed with Gwet’s AC2, was as follows: arterial-phase overall image quality, 0.712; arterial-phase hepatic artery clarity, 0.860; portal-phase overall image quality, 0.509; and portal-phase liver contrast, 0.348.

### 3.3. Optimal Iodine Dose Thresholds

ROC curve analysis identified the optimal iodine dose per body weight (mg I/kg) associated with a mean image quality score more than 4. At 100 kVp, the cutoff was 501.691 mg I/kg (AUC = 0.615 [95% CI, 0.525–0.705], *p* = 0.025); at 120 kVp, 599.145 mg I/kg (AUC = 0.596 [95% CI, 0.510–0.681], *p* = 0.036). For the portal venous phase, the optimal iodine dose was 517.650 mg I/kg (AUC = 0.628 [95% CI, 0.559–0.697], *p* = 0.001). In the arterial phase, the cutoff for hepatic artery clarity corresponding to a mean score more than 4 was 472.649 mg I/kg (AUC = 0.583 [95% CI, 0.418–0.749], *p* = 0.454) at 100 kVp and 592.175 mg I/kg (AUC = 0.713 [95% CI, 0.608–0.819], *p* = 0.001) at 120 kVp. In the portal phase, the cutoff for liver contrast predicting mean score more than 4 was 517.650 mg I/kg (AUC = 0.656 [95% CI, 0.583–0.729], *p* < 0.001).

For clinical applicability, these values were subsequently rounded to practical bands (500 mg I/kg at 100 kVp, 600 mg I/kg at 120 kVp, and 520 mg I/kg at portal phase), and corresponding sensitivity and specificity were recalculated. The corresponding sensitivity and specificity were 54.1% and 67.5%, 50.0% and 68.7%, and 54.7% and 63.0%, respectively, demonstrating comparable diagnostic performance to the statistically derived Youden-based cut-offs. These rounded thresholds were further converted to equivalent injection volumes for both contrast concentrations (270 and 350 mg I/mL) according to the current protocol, corresponding to approximately 1.85–2.2 mL/kg and 1.43–1.71 mL/kg, respectively (Table 3).

### 3.4. Quantitative Analysis

In the quantitative analysis, liver SNR was significantly higher in the HCIC group during both the arterial and portal phases (*p* < 0.001 for each). However, CNR of the aorta and portal vein did not differ significantly between groups. For HCC lesions, CNR of both arterial-phase and portal-phase also showed no significantly intergroup differences (Table 4).

### 3.5. Per-Patient Diagnostic Performance

A total of 323 patients with sufficient follow-up were included in the per-patient analysis (HCIC, *n* = 152; LCIC, *n* = 171). In the HCIC group, 26 had malignant lesions and 126 had benign lesions or no focal lesions. Pooled diagnostic performance in this group was as follows: sensitivity, 61.54% (32/52); specificity, 99.603% (251/252); positive predictive value (PPV), 96.970% (32/33); negative predictive value (NPV), 92.620% (251/271); and accuracy, 93.092% (283/304). In the LCIC group, 24 had malignant lesions and 147 had benign lesions or no focal lesions. Pooled per-patient diagnostic performance was: sensitivity, 64.58% (31/48); specificity, 99.660% (293/294); PPV, 96.88% (31/32); NPV, 94.516% (293/310); and accuracy, 94.737% (324/342). The area under the receiver operating characteristic curves did not differ significantly between groups (0.931 versus 0.947; *p* = 0.38; Table 5). No factor significantly affected per-patient diagnostic accuracy.

### 3.6. Per-Lesion Detectability and Diagnostic Performance

**Figure 3:**
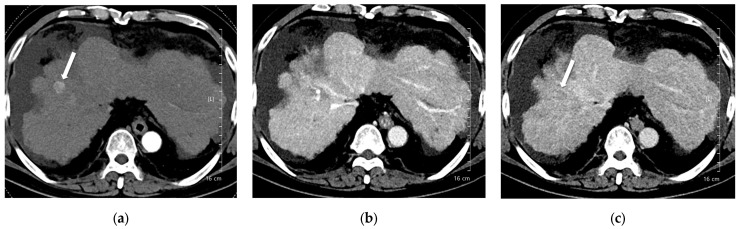
A 66-year-old man with hepatocellular carcinoma (HCC). CT was performed using high-concentration iodine contrast material (total iodine dose, 677.82 mg I/kg). Overall image quality in the arterial and portal phases was acceptable (mean scores more than 4): (**a**) A 15-mm enhancing nodule (arrow) is visible on the arterial phase at 120 kVp; (**b**) The nodule is inconspicuous on the portal phase; (**c**) Washout is evident on the delayed phase (arrow). Both reviewers diagnosed the lesion as HCC.

**Figure 4:**
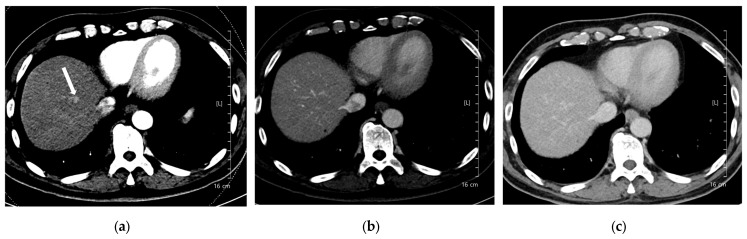
A 55-year-old man with hepatocellular carcinoma (HCC) on magnetic resonance imaging, characterized by diffusion restriction, high signal intensity on T2-weighted images, and washout. CT was performed using high-concentration iodine contrast material (total iodine dose, 584.14 mg I/kg). Overall image quality in the arterial and portal phases was acceptable. (**a**) A 7-mm enhancing nodule (arrow) is visible on the arterial phase at 100 kVp; (**b**,**c**) The nodule is inconspicuous on the portal and delayed phases. Both reviewers classified the lesion as benign (LI-RADS category 3).

**Figure 5:**
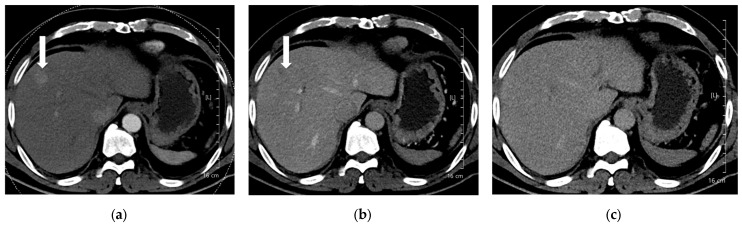
A 56-year-old man with hepatocellular carcinoma (HCC). CT was performed using low-concentration iodine contrast material (total iodine dose, 389.89 mg I/kg). Overall image quality in the arterial and portal phases was unacceptable (mean scores < 4): (**a**) A 17-mm enhancing nodule (arrow) is visible on the arterial phase at 120 kVp; (**b**) The nodule shows subtle washout on the portal phase (arrow); (**c**) The nodule is inconspicuous on the delayed phase. The two readers classified the lesion as LI-RADS 4 and LI-RADS 3, respectively.

**Figure 6:**
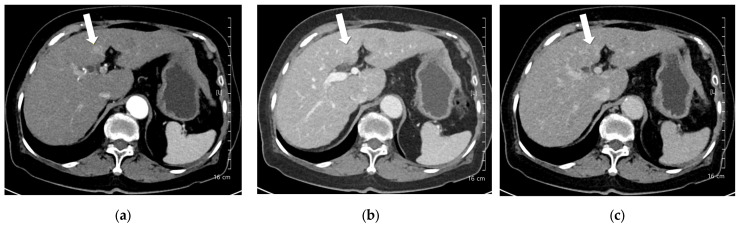
A 91-year-old woman with hepatocellular carcinoma (HCC). CT was performed using low-concentration iodine contrast material (total iodine dose, 518.4 mg I/kg). Overall image quality in the arterial and portal phases was acceptable. (**a**) An 8-mm enhancing nodule (arrow) is visible on the arterial phase at 100 kVp; (**b**,**c**) The nodule shows washout on the portal and delayed phases (arrows). Both reviewers classified the lesion as hepatocellular carcinoma.

A total of 168 focal liver lesions were identified in 93 participants (80 lesions in the high-concentration iodine group [*n* = 47] and 88 lesions in the low-concentration iodine group [*n* = 46]). Lesions comprised HCCs (*n* = 80), adenocarcinomas (*n* = 14), dysplastic nodules (*n* = 15), hemangiomas (*n* = 33), and regenerative nodules (*n* = 3). Lesion size was equivalent between the high- and low-concentration iodine groups (14.36 ± 14.72 mm versus 17.50 ± 12.27 mm, *p* = 0.134). Per-lesion detectability showed no significant difference between the groups (0.862 versus 0.909, *p* = 0.18; Table 6). Among detected lesions, the mean conspicuity score was significantly higher in the LCIC group than in the HCIC group (4.41 versus 4.07, *p* = 0.002) (Figure 3, Figure 4, Figure 5 and Figure 6).

Per-lesion diagnostic performance differed significantly between the two groups (0.669 versus 0.781; *p* = 0.02; Table 7). Lesion size significantly affected diagnostic performance (*p* < 0.001). The figure of merit did not differ significantly between the two groups (0.612 versus 0.633; mean difference, 0.021; 95% CI, −0.0922 to 0.1345; *p* = 0.710).

## 4. Discussion

Using low-concentration iodine contrast (270 mg I/mL) with modern MDCT and model-based iterative reconstruction yielded image quality and diagnostic capability non-inferior to conventional high-concentration contrast (350 mg I/mL). Although liver SNR was significantly lower with the LCIC protocol, lesion conspicuity remained clinically acceptable, and lesion detectability was similar between two groups. In addition, ROC analysis identified voltage-specific iodine-dose threshold that preserved adequate image quality, with lower thresholds at 100 kVp (500 mg I/kg) than at 120 kVp (600 mg I/kg).

These findings accord with prior studies showing that iodine-dose reduction strategies—lowering tube voltage, applying iterative or spectral CT techniques (e.g., virtual monoenergetic imaging), and using deep learning-based reconstruction—can preserve diagnostic image quality despite reduced iodine load [3,4,5,12,13,16,17,22]. Most of those studies reduced iodine dose by lowering the injection volume of standard high-concentration contrast media (350 mg I/mL). In contrast, we reduced contrast concentration (270 mg I/mL), which maintained image quality and may lessen contrast-related complications such as nephrotoxicity. Recent investigations using deep learning-based reconstruction likewise reported non-inferior image quality with 270 mg I/mL protocols [12,13]. Extending these observations, we showed that, under routine clinical conditions with modern MDCT and model-based iterative reconstruction, low-concentration protocols achieve diagnostically acceptable performance without meaningful differences from high-concentration protocols. In the present study, both arterial and portal phases were analyzed, and the findings were consistent with Yanaga et al. [1]. Adequate image quality was achieved with approximately 500 mg I/kg (100 kVp) and 600 mg I/kg (120 kVp) in the arterial phase, while about 520 mg I/kg was sufficient in the portal phase, indicating that dual-energy CT is not essential when iodine dose is properly adjusted for tube voltage and body weight. The equivalent injection volume derived from these values facilitates straightforward clinical application by allowing technologists to apply these thresholds in routine workflow without additional adjustment. In addition to reduced iodine exposure and injection pressure, these results emphasize the practical feasibility of voltage-and weight-adapted LCIC protocols for safe, cost-effective routine liver CT.

Beyond these overall findings, some phase- and subgroup-specific results are worth discussing to aid interpretation. Qualitative arterial-phase analysis showed no meaningful differences between the HCIC and LCIC groups in overall image quality or hepatic artery clarity. In the portal phase, the LCIC group exhibited significantly lower overall image quality and parenchymal contrast, yet both metrics met the predefined non-inferiority margin of −0.5. This pattern aligns with reports that portal-phase enhancement depends on total iodine delivery and concentration, whereas arterial enhancement is less sensitive to iodine concentration [3,4].

Quantitatively, higher vascular CNR is physiologically expected in the HCIC group. However, in some LCIC subgroups, vascular CNR was non-significantly higher, likely because lower background fat enhancement increased HU differences and thus CNR despite the reduced iodine dose. Among patients with HCC, arterial-phase CNR tended to be non-significantly higher in the HCIC group, consistent with the expectation that higher iodine concentration improves tumor conspicuity in the arterial phase. These trends were more pronounced at 100 kVp, likely because iodine attenuation at this setting is closer to its k-edge (33.2 keV), enhancing the photoelectric effect and increasing sensitivity to iodine concentration [24]. However, interpretation was limited by the very small sample size of HCIC lesions (*n* = 4), including two lesions with unusually strong arterial enhancement that resulted in high CNR values. Nevertheless, subgroup analyses stratified by tube voltage did not reach statistical significance, and no significant intergroup differences were observed in the portal phase.

Per-patient diagnostic performance remained high and consistent across protocols and clinical subgroups. At the lesion level, overall detectability was similar between groups (0.862 versus 0.909, *p* = 0.18), whereas the LCIC group unexpectedly showed higher diagnostic accuracy (0.669 versus 0.781, *p* = 0.02). It could reflect sample heterogeneity rather than true superiority. Given the lesion conspicuity scores (4.07 versus 4.41, *p* = 0.002), a larger proportion of low-conspicuity HCCs may have been included in the HCIC cohort. Thus, this finding should not be interpreted as evidence of LCIC superiority but as indicating equivalent diagnostic reliability to HCIC. As expected, lesion size significantly influenced detectability and characterization (*p* for factor < 0.001), and the apparent group difference likely reflects lesion-size distribution rather than iodine concentration. Taken together, these observations suggest that lowering iodine concentration does not compromise diagnostic reliability at either the patient or lesion level and align with recent studies demonstrating preserved diagnostic accuracy under reduced-iodine protocols [13,14].

This study has several limitations. First, the retrospective design limited control over confounding and patient selection. Sequential enrollment could introduce temporal bias. Although efforts were made to balance baseline characteristics, prospective validation is warranted to confirm generalizability. Second, tube-voltage distribution differed between the two contrast groups. We mitigated this with subgroup analyses, which consistently showed no significant differences, supporting the robustness of the primary results. Third, per-lesion diagnostic performance was evaluated using a composite reference standard that included imaging follow-up, which may have introduced verification bias, particularly for lesions without histopathologic confirmation. However, in current clinical practice, HCC can be reliably diagnosed by CT and MRI without biopsy according to established guidelines [25,26], and therefore this strategy was considered appropriate for the present study. Fourth, the between-subject rather than intraindividual design may have allowed subtle inter-patient variability in vascular physiology or hepatic parenchymal characteristics to influence imaging outcomes. Fifth, ROC-derived iodine dose thresholds have modest AUC values. This finding is likely due to the influence of multiple patient- and technique-related factors on image quality, in addition to the amount of administered iodine. Sixth, given that the risk of intravenous contrast-induced acute kidney injury (CI-AKI) is very low in patients with baseline serum creatinine levels below 1.5 mg/dL, our study did not include a dedicated AKI analysis within 48–72 h after contrast administration. Future prospective research incorporating renal function assessments is warranted to clarify potential subclinical renal effects. In addition, dose–response data derived from intra-arterial angiography studies [2,27] may not be directly applicable to contemporary intravenous CT settings due to differences in hemodynamic and contrast delivery characteristics. Seventh, the dual-reader blinded evaluation represents a methodological strength of this study. However, the interobserver agreement in the portal phase (AC2 = 0.35) was relatively low, indicating variability in subjective assessment of subtle contrast differences. This finding may reflect intrinsic challenges in evaluating moderate parenchymal enhancement during the portal phase, where lesion conspicuity tends to be lower than in the arterial phase. In addition, the exclusion of non-detectable lesions from the contrast-to-noise ratio (CNR) analysis could have introduced selection bias, as such lesions represent true detection failures in clinical practice. Including these cases in an intention-to-detect framework in future studies may improve transparency and better reflect real-world diagnostic performance.

## 5. Conclusions

Low-concentration iodine contrast yields diagnostic outcomes non-inferior to high-concentration contrast agents in hepatic multiphase CT using modern MDCT systems. Tube voltage significantly influences the iodine dose required to maintain optimal arterial-phase image quality. These findings support protocol optimization for safer, more individualized liver CT imaging.

## Figures and Tables

**Table 1:** Demographics of the Study Population.

	HCIC CT (*N* = 179)	LCIC CT (*N* = 190)	*p*
Sex, *n* (men:women)	127:52	137:53	0.806
Age, years			
Men	61.755 ± 11.514 (34–86)	61.839 ± 12.080 (29–88)	0.954
Women	63.654 ± 11.800 (42–88)	62.472 ± 13.499 (27–91)	0.634
Underlying disease, % (*n*/*N*)			
Chronic hepatitis B	50.279 (90/179)	54.737 (104/190)	0.391
Chronic hepatitis C	5.587 (10/179)	4.737 (9/190)	0.712
Alcoholic liver disease	6.145 (11/179)	4.737 (9/190)	0.55
Cryptogenic	35.754 (64/179)	34.211 (65/190)	0.756
Laboratory findings			
Albumin, g/dL	4.317 ± 0.586 (2.0–5.3)	4.259 ± 0.591 (2.1–5.1)	0.346
Total bilirubin, mg/dL	1.246 ± 2.630 (0.24–30.1)	1.214 ± 1.690 (0.18–17.14)	0.89
INR	1.100 ± 0.183 (0.89–2.51)	1.142 ± 0.494 (0.90–7.17)	0.279
Platelet count, ×10^3^/mm^3^	158.550 ± 67.437 (27–375)	157.810 ± 69.179 (30–446)	0.918
AFP, ng/mL	24.002 ± 146.684 (1–957)	521.124 ± 4952.931 (0.9–50,000)	0.184
Body weight, kg	68.171 ± 15.089 (28–124.5)	69.512 ± 13.630 (40.3–123.8)	0.371
Contrast amount, mg I/kg	626.189 ± 124.592 (337.35–1375)	468.697 ± 74.053 (261.71–736.97)	<0.001
Body mass index, kg/m^2^	24.822 ± 4.329 (11.65–39.46)	25.336 ± 4.049 (14.48–39.56)	0.239
DLP, mGy·cm	976.700 ± 450.728 (333–3392)	949.650 ± 454.449 (401–4288)	0.566
Effective dose, mSv	14.651 ± 6.761 (5.00–50.88)	14.245 ± 6.817 (6.02–64.32)	0.566

Note: Values are mean ± standard deviation; ranges are minimum–maximum. *n* indicates the number of patients with the characteristic; *N*, the total cohort. Units are given after each variable. Effective dose was calculated as dose-length product × 0.015 mSv/(mGy·cm), the standard coefficient for abdominal CT. Abbreviations: HCIC CT, high-concentration iodine contrast CT; LCIC CT, low-concentration iodine contrast CT.

**Table 2:** Qualitative image-quality analysis comparing high-concentration iodine contrast CT (HCIC CT) and low-concentration iodine contrast CT (LCIC CT).

Outcome	HCIC CT (*n* = 179)	LCIC CT (*n* = 190)	*p*	Mean Difference
Arterial phase				
Overall image quality				
Overall (*n* = 369)	4.450 ± 0.462 [4.382, 4.518]	4.439 ± 0.477 [4.371, 4.508]	0.834	−0.010 [−0.107, 0.086]
120 kVp (*n* = 172)	4.391 ± 0.479 [4.331, 4.472]	4.426 ± 0.579 [4.224, 4.629]	0.714	0.035 [−0.154, 0.224]
100 kVp (*n* = 197)	4.646 ± 0.340 [4.539, 4.754]	4.442 ± 0.454 [4.370, 4.514]	0.008	−0.204 [−0.354, −0.054]
Hepatic artery clarity				
Overall (*n* = 369)	4.802 ± 0.380 [4.746, 4.858]	4.834 ± 0.357 [4.783, 4.885]	0.397	0.033 [−0.043, 0.108]
120 kVp (*n* = 172)	4.754 ± 0.415 [4.684, 4.824]	4.647 ± 0.516 [4.467, 4.827]	0.204	−0.107 [−0.272, 0.058]
100 kVp (*n* = 197)	4.963 ± 0.132 [4.922, 5.005]	4.875 ± 0.299 [4.828, 4.922]	0.066	−0.088 [−0.183, 0.006]
Portal phase				
Overall image quality	4.430 ± 0.443 [4.365, 4.495]	4.337 ± 0.371 [4.284, 4.390]	0.028	−0.093 [−0.177, −0.010]
Liver contrast rating	4.536 ± 0.407 [4.476, 4.596]	4.384 ± 0.391 [4.328, 4.440]	<0.001	−0.152 [−0.234, −0.070]

Note: Data are presented as mean ± standard deviation, with 95% confidence intervals in brackets. Mean difference was calculated as LCIC CT − HCIC CT; negative values indicate superior performance of the HCIC CT group.

**Table 3:** Rounded cutoff values for iodine dose and corresponding injection volumes predicting diagnostically acceptable image quality (Likert score ≥ 4) based on receiver operating characteristic (ROC) analysis.

	* Rounded Cutoff (mg I/kg)	Sensitivity (%)	Specificity (%)	Injection Volume (mL/kg)	
				HCIC (350 mg I/mL)	LCIC (270 mg I/mL)
Arterial phase					
100 kVp	500	54.1	67.5	1.43	1.85
120 kVp	600	50.0	68.7	1.71	2.22
Portal phase	520	54.7	63.0	1.49	1.93

* Cutoff values were rounded for clinical applicability. Injection volumes were derived by dividing the iodine dose (mg I/kg) by the contrast concentration (mg I/mL) for each protocol (HCIC = 350 mg I/mL; LCIC = 270 mg I/mL).

**Table 4:** Quantitative analysis of image-quality parameters in HCIC CT versus LCIC CT.

Parameter	HCIC CT (*n* = 179)	LCIC CT (*n* = 190)	*p*
Liver SNR			
Arterial phase			
Overall (*n* = 369)	8.096 ± 1.828	6.843 ± 1.859	<0.001
100 kVp (*n* = 197)	8.323 ± 1.505	6.737 ± 1.736	<0.001
120 kVp (*n* = 172)	8.029 ± 1.913	7.329 ± 2.312	0.069
Portal phase	12.176 ± 2.788	10.738 ± 2.624	<0.001
Aorta CNR			
Overall (*n* = 369)	61.411 ± 16.796	63.813 ± 16.185	0.163
100 kVp (*n* = 197)	73.840 ± 19.365	65.770 ± 16.065	0.007
120 kVp (*n* = 172)	57.718 ± 14.038	54.838 ± 13.673	0.283
Portal vein CNR	32.418 ± 6.927	32.515 ± 7.111	0.895
HCC CNR			
Arterial phase			
Overall (detected HCC, *n* = 54)	4.342 ± 2.535	2.907 ± 2.857	0.057
100 kVp (*n* = 29)	5.190 ± 1.822	2.652 ± 2.797	0.093
120 kVp (*n* = 25)	4.188 ± 2.648	5.030 ± 2.954	0.614
Portal phase (detected HCC, *n* = 58)	−1.006 ± 3.065	−1.585 ± 2.477	0.43

Notes: Of 80 hepatocellular carcinomas (HCCs), CNR was measured in 54 lesions visible in the arterial phase (100 kVp, *n* = 29; 120 kVp, *n* = 25). In the portal phase, CNR was assessed in 58 visible lesions. Abbreviations: HCIC CT, high-concentration iodine contrast CT; LCIC CT, low-concentration iodine contrast CT; SNR, signal-to-noise ratio; CNR, contrast-to-noise ratio.

**Table 5:** Comparison of per-patient diagnostic accuracy between HCIC CT and LCIC CT (*n* = 323).

Subgroup	HCIC CT (*n* = 152)	LCIC CT (*n* = 171)	*p*	*p* for Factor
	Diagnostic Accuracy	Diagnostic Accuracy		
All patients (*n* = 323)	0.931 [0.896–0.955]	0.947 [0.918–0.967]	0.38	
120 kVp (*n* = 154)	0.934 [0.896–0.958]	0.942 [0.836–0.981]	0.82	0.80
100 kVp (*n* = 169)	0.917 [0.798–0.968]	0.948 [0.916–0.969]	0.26	
No ascites (*n* = 307)	0.928 [0.891–0.952]	0.951 [0.921–0.97]	0.83	0.84
Ascites present (*n* = 16)	1 [1,1]	0.889 [0.648–0.972]	0.23	
No portal hypertension (*n* = 237)	0.937 [0.896–0.962]	0.952 [0.918–0.973]	0.46	0.34
Portal hypertension present (*n* = 86)	0.915 [0.832–0.959]	0.933 [0.859–0.97]	0.64	
BMI < 25.0 (*n* = 167)	0.922 [0.870–0.954]	0.946 [0.900–0.972]	0.36	0.60
BMI ≥ 25.0 (*n* = 156)	0.942 [0.888–0.971]	0.948 [0.904–0.973]	0.81	

Note: Diagnostic accuracy was estimated using generalized estimating equations (binomial distribution; logit link). Values are reported with 95% confidence intervals in brackets. Subgroup analyses evaluated performance across patient characteristics and imaging parameters.

**Table 6:** Comparison of per-lesion detectability between HCIC CT and LCIC CT (*n* = 168 lesions).

	HCIC CT (*n* = 80)	LCIC CT (*n* = 88)	*p*	*p* for Factor
	Detectability	Detectability		
All lesions (*n* = 168)	0.862 [0.800–0.908]	0.909 [0.857–0.943]	0.18	
120 kVp (*n* = 76)	0.864 [0.794–0.912]	0.900 [0.676–0.975]	0.66	0.761
100 kVp (*n* = 92)	0.857 [0.676–0.945]	0.910 [0.855–0.946]	0.39	
Lesion size < 20 mm (*n* = 129)	0.838 [0.767–0.891]	0.869 [0.798–0.917]	0.49	<0.001
Lesion size ≥ 20 mm (*n* = 39)	1.000 [1.000–1.000]	1.000 [1.000–1.000]	0.98	

Note: Lesion detectability was estimated using GEE with a binomial distribution and a logit link. Values are detectability rates with 95% confidence intervals in brackets. Subgroups were defined by tube voltage and lesion size.

**Table 7:** Comparison of per-lesion diagnostic performance between HCIC CT and LCIC CT (*n* = 168 lesions).

	HCIC CT (*n* = 80)	LCIC CT (*n* = 88)	*p*	*p* for Factor
	Diagnostic Accuracy	Diagnostic Accuracy		
All lesions (*n* = 168)	0.669 [0.592–0.737]	0.781 [0.713–0.836]	0.02	
120 kVp (*n* = 76)	0.674 [0.590–0.749]	0.850 [0.624–0.951]	0.12	0.71
100 kVp (*n* = 92)	0.643 [0.454–0.796]	0.772 [0.699–0.831]	0.15	
Lesion size < 20 mm (*n* = 129)	0.618 [0.533–0.695]	0.696 [0.608–0.772]	0.19	<0.001
Lesion size ≥ 20 mm (*n* = 39)	0.958 [0.756–0.994]	0.963 [0.864–0.991]	0.92	

Note: Diagnostic accuracy was evaluated using generalized estimating equations (GEE; binomial distribution, logit link). Multivariable GEE was used to assess the effect of the main independent variable (contrast group) with covariates (CT type and lesion size).

## Data Availability

The data presented in this study are available on request from the corresponding author. The data are not publicly available due to privacy and ethical restrictions, as the dataset contains patient-related information, including de-identified Likert-scale image quality scores, lesion lists, and iodine dose values (mg I/kg).

## Supplementary Materials

The following supporting information can be downloaded at: https://www.mdpi.com/article/10.3390/diagnostics15233026/s1; Figure S1: CT images acquired using SOMATOM Definition Flash.; Figure S2: CT images acquired using SOMATOM Force.

## Abbreviations

The following abbreviations are used in this manuscript:

AUC	area under the curve
CARE	combined applications to reduce exposure
CCC	cholangiocarcinoma
CI	confidence interval
CNR	contrast-to-noise ratio
CT	computed tomography
DE	dual-energy
DLP	dose-length product
GEE	generalized estimating equations
HCC	hepatocellular carcinoma
HCIC	high-concentration iodine contrast
HU	Hounsfield unit
LCIC	low-concentration iodine contrast
MRI	magnetic resonance imaging
NPV	negative predictive value
PPV	positive predictive value
ROC	receiver operating characteristic
ROI	region of interest
SD	standard deviation
SNR	signal-to-noise ratio
