# Supplementary material for: Low- Versus High-Concentration Iodine Contrast for Hepatic Multiphase CT in Chronic Liver Disease: Image Quality, Lesion Detectability, and Iodine Load Reduction with Modern MDCT—A Retrospective Non-Inferiority Study
Source: Diagnostics (Basel). 2025 Nov 27;15(23):3026. doi: 10.3390/diagnostics15233026 (PMC12691093; doi:10.3390/diagnostics15233026)
Supplement: Supplementary file 1 [file diagnostics-15-03026-s001.zip › diagnostics-4001809-supplementary.pdf]

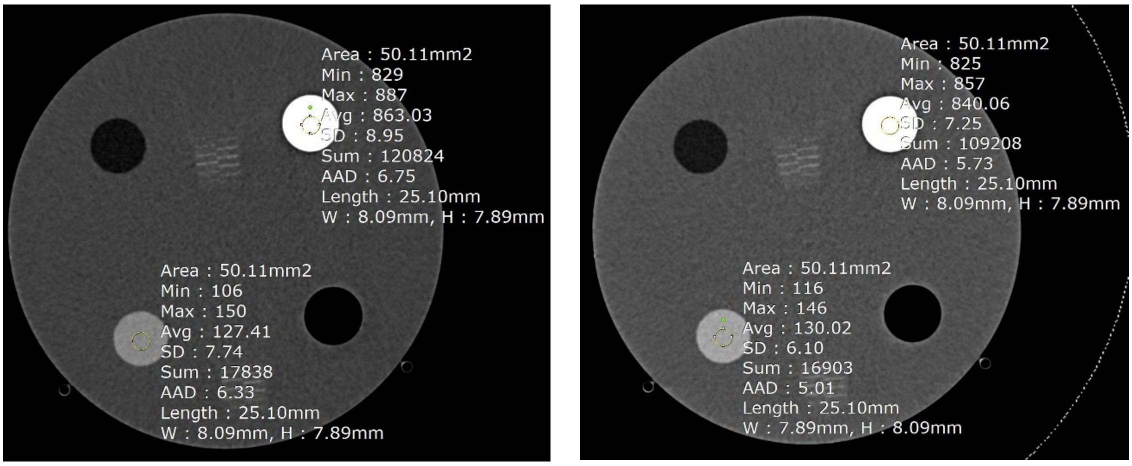

(**a**) (**b**)

Figure S1. CT images acquired using SOMATOM Definition Flash. (a) Image obtained with 120 kVp single-energy mode. (b) Blended image reconstructed from dual-energy acquisition. The Hounsfield units of the two images are nearly identical.

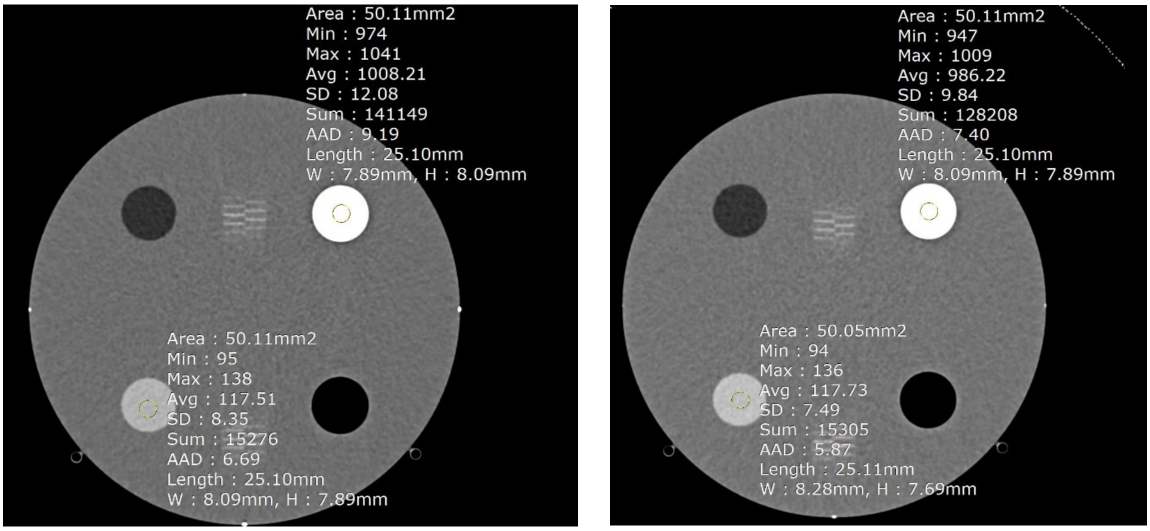

(**a**) (**b**)

Figure S2. CT images acquired using SOMATOM Force (a) Image obtained with 100 kVp single-energy mode. (b) Blended image reconstructed from dual-energy acquisition. The Hounsfield units of the two images are nearly identical.
